# Unexpected but welcome. Artificially selected traits may increase fitness in wild boar

**DOI:** 10.1111/eva.12383

**Published:** 2016-05-17

**Authors:** Domenico Fulgione, Daniela Rippa, Maria Buglione, Martina Trapanese, Simona Petrelli, Valeria Maselli

**Affiliations:** ^1^ Department of BiologyUniversity of Naples Federico IINaplesItaly

**Keywords:** fitness, interdemic selection, *MC*1*R* gene, pig, reproduction, wild boar

## Abstract

Artificial selection affects phenotypes differently by natural selection. Domestic traits, which pass into the wild, are usually negatively selected. Yet, exceptionally, this axiom may fail to apply if genes, from the domestic animals, increase fertility in the wild. We studied a rare case of a wild boar population under the framework of Wright's interdemic selection model, which could explain gene flow between wild boar and pig, both considered as demes. We analysed the *MC*1*R* gene and microsatellite neutral loci in 62 pregnant wild boars as markers of hybridization, and we correlated nucleotide mutations on *MC*1*R* (which are common in domestic breeds) to litter size, as an evaluation of fitness in wild sow. Regardless of body size and phyletic effects, wild boar sows bearing nonsynonymous *MC*1*R* mutations produced larger litters. This directly suggests that artificially selected traits reaching wild populations, through interdemic gene flow, could bypass natural selection if and only if they increase the fitness in the wild.

## Introduction

Human impact on natural populations can affect the phenotype of domestic forms through at least three distinct processes. First and foremost, domestication targets traits, that are beneficial to humans (Rauw et al. 1998). Secondly, small captive populations are exposed to genetic drift and inbreeding (Willoughby et al. 2015). Thirdly, wild individuals may have experienced unusual natural selection pressures (Hutchings and Fraser 2008), becoming attractive to humans even before domestication (Lega et al. 2015).

The fixation of novel phenotypic variants in domestic animals is possible, although it is usually counterfeited by breeding between wild and domestic individuals (Marshall et al. 2014), and the opposite is far less common (Hostetler et al. 2013). Yet, there is good evidence for gene flow between pig, sheep, goat, cattle and their wild relatives, where they can be found in sympatry (Larson and Burger 2013; Marshall et al. 2014), as a product of intentional or accidental process either. Such interbreeding is generally seen as a potential threat to wild populations (Allendorf et al. 2001; Randi 2008). Yet, occasionally, the fixation of domestic phenotypic traits in wild individuals may play an important role in evolution (Allendorf et al. 2001).

As they are primarily raised for meat, most of the world's pig breeds (*Sus scrofa*) were selected to increase traits such as growth rate and fertility. Strong selection has resulted in unintentional reduction in brain mass (Maselli et al. 2014), limb length and flight distance. By using genome‐wide assays, Goedbloed and co‐authors (Goedbloed et al. 2013) speculated that genetic introgression from pig breeds could alter the fertility in wild forms. This speculation appears often in the scientific literature (Young 1995), yet it was never demonstrated empirically. Frequent genetic introgression from domestic pigs may lead to either hybrid vigour or maladaptation to natural environment (Verhoeven et al. 2010). This means that free‐living pigs may represent a significant threat to the genetic integrity of wild boar populations (Marshall et al. 2014), whose likelihood to fix domestic traits in the wild‐type is thus counterfeited by negative selection on correlated traits.

The Southern Italian wild boar population is an interesting model species in which contact between wild and domestic individuals is common, due to traditional farming practices (Randi 2005; Maselli et al. 2014). This provides a unique opportunity to study what happens when artificially selected traits are fixed in the wild‐type genome and evolve.

Here, we demonstrate as high fertility in pigs can introgress into the wild boar, via hybridization, affecting fitness. This introgression is so pervasive as to overbalance the negative effects of additional artificially selected traits that would otherwise be detrimental to survival (Marshall et al. 2014). Moreover increasing of knowledge about wild boar fertility could be useful for the development of suitable management strategy.

## Materials and methods

### Sampling and pregnant individuals' anatomy

Gathering data on litter size of wild boar are not easy to obtain because field observations can be inaccurate or incorrect, so hunted animals (especially pregnant) are successfully used in these studies (Gaillard et al. 1987; Fernández‐Llario et al. 1999; Fernández‐Llario and Mateos‐Quesada 2005).

Our samples were collected in the Cilento, Vallo of Diano and Alburni National Park (CVD, South Italy, 181 000 ha), during legal hunts in accordance with Italian National laws (*157/92 and 394/91 Laws*). Moreover, all field protocols were approved by the Ministry of Environment (ISPRA, prot. n 24581 20/07/2014). We joined a demographic control plan of wild boar in the CVD, where this species represents a demographic and ecologic problem, and collected data on sows shot by specialized hunters.

All culled animals (*n* = 500), both male and female (*n* = 228, *n* = 272, respectively), were checked in order to identify domestic variants according to body morphology and coat colour. The reproductive status of 272 females (pregnant, lactating or nonbreeding, neither pregnant nor lactating) was recorded and the gravid uterus was removed during necropsy. Within this sampling, we extracted 62 pregnant sows belonging to 18 free‐ranging populations living in CVD. We considered pregnant females with gestational age of at least two months, when the potential fertility rate is roughly the live birth rate, and the probability of prenatal mortality is minimal (Náhlik and Sándor 2003). In any case, foetuses prematurely dead or absorbed were excluded from the count. We took the number of foetuses per litter as litter size (Fonseca et al. 2004). Females were weighed (± 1 kg) and assigned to age classes by analysing the tooth eruption and replacement patterns (Baubet et al. 1994; Pedone et al. 1995).

From each pregnant female, we extracted total genomic DNA by using QIAamp DNA Mini Kit (QIAGEN, Valencia, CA), according to the manufacturer's instructions.

### Population genetics of neutral loci

To assess the genetic structure of the wild boar samples, a microsatellite analysis was performed for nine polymorphic loci: SW461, SW2532, SW2021, S0063, S0174, S0175, S0176, S0177, S0179 (details at http://www.thearkdb.org). Polymerase chain reaction amplifications were carried out in 10 *μ*L final volumes containing 20 ng of genomic DNA, 0.50 *μ* m of each primer, 10× PCR buffer, 0.2 U Taq polymerase (DreamTaq; Fermentas, Vilnius, Lithuania), 0.25 mm each dNTP. The thermocycler profile started with an initial denaturation step at 94°C for 3 min, followed by 35 cycles at 94°C for 30 s, T annealing at 50‐55°C for 1 min, 72°C for 1 min followed by 72°C for 5 min. A negative control was run with each round of PCR.

Polymorphism of microsatellite was determined using one of each pair primers end‐labelled, with a fluorescent dye group (FAM and HEX; MWG Biotech, Ebersberg, Germany), and an internal size standard LIZ500. Amplified DNA fragments were electrophoresed using an ABI 3100 automated sequencing instrument (Applied Biosystems, Perkin‐Elmer/Cetus, Norwalk, CT, USA) sequencer, and their genotypes were analysed with GeneMarker Software (Softgenetics, State College, PA, USA), version 1.9.

Genetic differentiation was analysed using global *F*_ST_ estimates, calculated in GENALEX 6.5 (Peakall and Smouse 2012). Significance of estimates was based on 999 permutations of the dataset. *F*_ST_ was calculated for all loci in wild‐type (E^+^) and mutate samples.

### mtDNA sequence analysis

In order to ascribe wild boars to mtDNA haplogroups, we analysed 652‐bp fragment of the control region for all samples, using the primers H16108 and L15387 (Watanobe et al. 2001; Larson et al. 2005; Maselli et al. in press).

PCR products were sequenced in both directions using the BigDye Terminator Kit on an ABI 3100 automated sequencer (Applied Biosystems, Foster City, CA).

Sequences were aligned with already published GenBank sequences sampled worldwide, chosen as to represent the current genetic diversity of Western Eurasia (Larson et al. 2005).

Pairwise genetic distances among samples were computed using Tamura‐Nei algorithm with the software Geneious 5.5 (Drummond et al. 2011).

We used phylogenetic logistic regression (Ives and Garland 2010) to test the hypotheses that genetic relatedness (mtDNA) predicts difference between sows.

### MC1R gene analysis for inbreeding and domestication inference

Modern domestic animal species display a bewildering diversity in coat colour, and the melanocortin receptor 1 (MC1R) locus is most consistently polymorphic, having been previously documented and associated with coat colour variation in horses, cattle, foxes, pigs, sheep, dogs and chickens (Cieslak et al. 2011). In pigs, domestication and subsequent selective pressures produced a great variety of coat colours in different regions and breeds because of different human needs or cultural preferences (Larson and Burger 2013). The wild‐type of melanocortin‐1 receptor (MC1R) coat colour gene has almost exclusively been identified in wild boars (Fajardo et al. 2008; Fang et al. 2009; Canu et al. in press) and its mutations have been used to detect wild/domestic hybrids (Koutsogiannouli et al. 2010; Frantz et al. 2013; Fontanesi et al. 2014).

In a recent paper about MC1R in Eurasian wild boar, it was detected a lower of polymorphism except for regions where pigs are often reared in a semi‐free conditions and may cross‐breed with the wild form (Canu et al. in press). According to these authors, introgression may reach high levels at very local scale, and/or that intentional hybridization in captivity may be an important source of introgression.

The entire coding region of MC1R gene was amplified and sequenced by using primer combinations (Maselli et al. 2014). The relative frequencies of synonymous and nonsynonymous substitutions were calculated using the Nei‐Gojobori method in MEGA software, version 4 (Nei and Gojobori 1986; Tamura and Nei 1993). Standard errors were estimated with bootstrap (500 replicates). The magnitude of pig introgression was estimated from both synonymous substitution rates (assumed to be neutral) and nonsynonymous substitution rates. Summary statistics involving coding regions included numbers of synonymous (d*S*) and nonsynonymous (d*N*) substitutions, were calculated using the KaKs_Calculator1.2 software (Zhang et al. 2006). KaKs_calculator 2.0 was run according to the MLWL method.

In order to investigate the correlation between genetic data and litter size we first calculated a genetic index (GI). The GI is the PC1 obtained performing a principal component analysis (PCA) using all mutation data on MC1R gene. Since the genetic variables were likely to be correlated, a PCA with subsequent varimax rotation was applied in order to reduce them to a smaller number of independent factors (Sokal and Rohlf 1995). Following the recommendations of Aspey and Blankenship (1977) and Bauer (1986), for interpretation only factors with eigenvalues ≥ 1 were extracted (Kaiser criterion) and only factors loadings ≥ 0.45 were considered to be meaningful. The GI was regressed against litter size.

## Results

**Figure 1:**
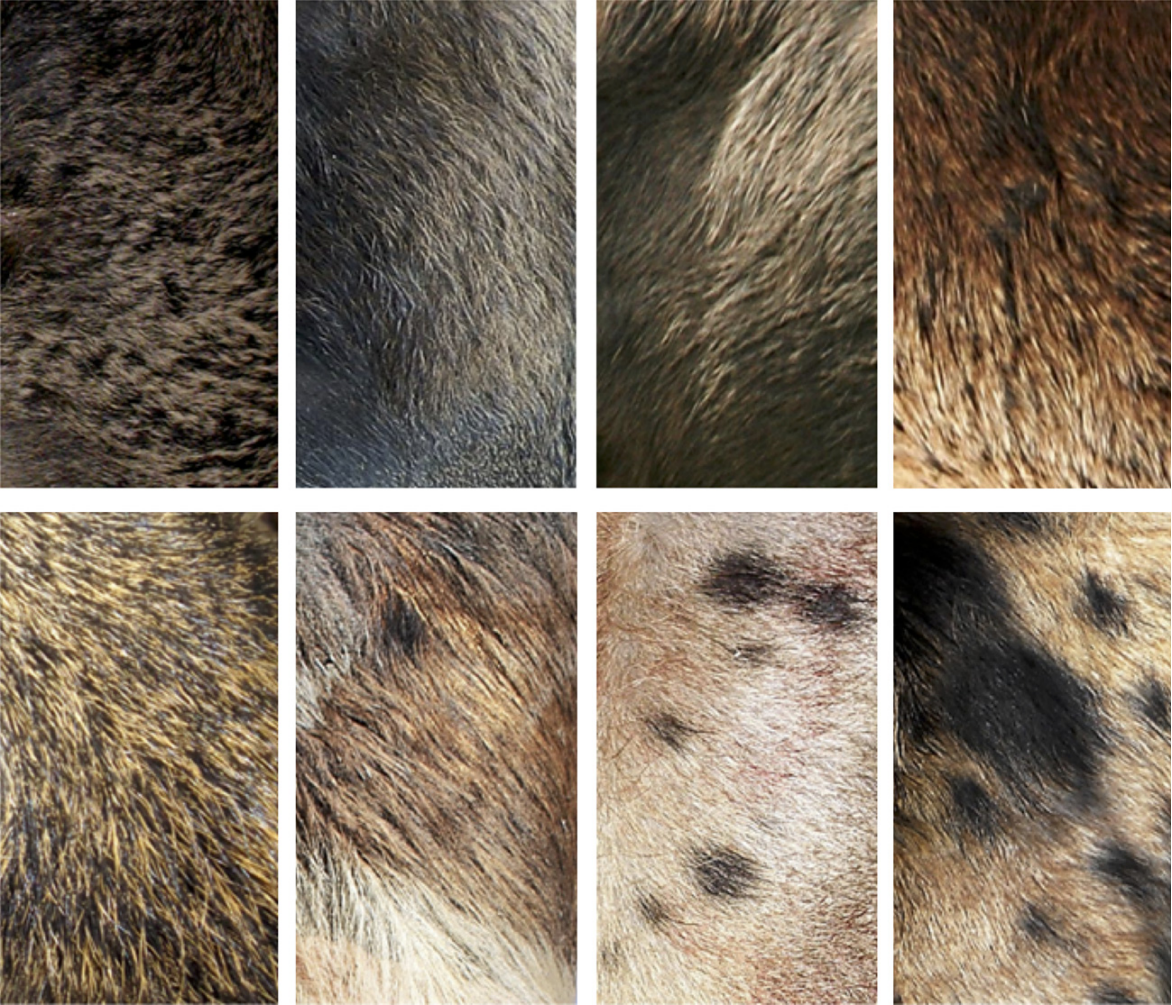
Phenotypic variations of the coat colour easily recognizable in some specimens belonging to Southern Italian wild populations of *Sus scrofa*.

We sampled 62 pregnant sows culled according to the program for demographic control developed in the CVD. It is easy to come across wild boar individuals bearing typically pig‐like features (i.e. floppy ears, curly tail and straight frontal bones) in CVD. In addition, the classic reddish/brown coat colour of wild boar is often replaced by red, spotted, black and grey uniform pelage there (Fig. 1). Our population model predicts that the rate of divergence at neutral loci should be lower according to hybridization among different forms and between wild boars and putative hybrids. Our data are fully consistent with this prediction, pairwise *F*_ST_ = 0.059 ± 0.024 (mean ± SE; *n* = 62) between our samples revealing no differentiation and consistent gene flow.

According to the matrilinear genetic analyses, our studied sows belong to the European, Italian and Asian clades (82.3%, 6.5% and 11.3%, respectively, mtDNA). Litter size was 6.16 ± 1.68 (mean ± SD; *n* = 62), ranging from three to 10 foetuses.

MC1R gene sequences revealed 13 different alleles (named Type 1 to 13; see supporting information Table S1), three of them were previously described elsewhere (Fang et al. 2009). A high proportion of samples belonged to the wild‐type (61.0% E^+^), whereas the remaining sows showed allelic variation, involving synonymous, and nonsynonymous mutations in MC1R, as well as deletions. The high degree of heterozygosity (63.6%) was not surprising for a wild population, especially considering that coat colour undergoes strong selection. In fact, MC1R gene was under negative purifying selection (Fisher exact test, d*N*/d*S* = 0.046; *P* ≪ 0.001).

We grouped sows in relation to mutations in their MC1R sequences. Individuals with mutations (both d*S* and d*N*) are more productive than the wild‐type (mean number of piglets per litter: samples with mutation = 7.06, wild‐type = 5.03). This difference is statistically significant (anova,* F* = 21.98, df = 45, *P* ≪ 0.001). This result does not depend on the effect of body size (ancova,* F* = 16.25, df = 43, *P* ≪ 0.001). The interaction between wild boar size and litter size is not significant (ancova,* F* = 1.36, df = 43, *P* = 0.25).

As compared to the wild‐type, sows bearing one synonymous mutation were not statistically more productive (mean number of piglets per litter in sows with one synonymous mutation = 5.70, *n* = 8). Only two samples, bearing two synonymous mutations, had higher productivity as compared to the others (mean number of piglets per litter in sows with two synonymous mutation = 8.37). Overall, synonymous mutations, neutral nucleotide changes, did not correlate with higher productivity (*F* = 1.764, *P* = 0.191, df = 45).

Nonsynonymous mutations were associated to higher productivity (mean number of piglets per litter: samples with nonsynonymous mutations = 7.70, *n* = 12; samples with synonymous mutations and wild‐type = 5.23, *n* = 35). These differences were statistically significant (anova,* F* = 22.97, *P* << 0.001, df = 45) and independent from body size (ancova,* F* = 23.16, *P* << 0.001, df = 45). The interaction between sow size and productivity was not significant when sows were partitioned according to the presence of nonsynonymous mutations in their MC1R sequence (ancova,* F* = 0.024, *P* = 0.878, df = 43).

The test of phylogenetic logistic regression still confirmed a significant difference in production, among sows, independently from phylogenetic signal (*z* value = −3.27, *P* = 0.001).

**Figure 2:**
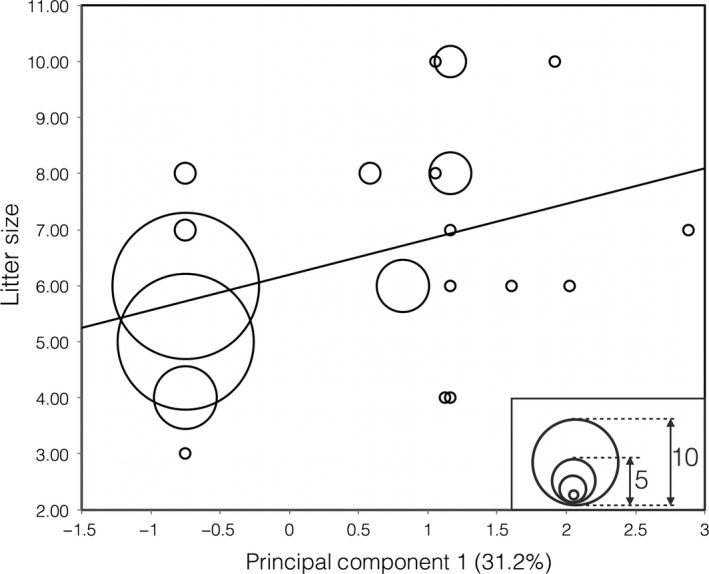
Increase litter size in wild boar related to the genetic variation on *MC*1*R* codogene. Mutations, and their mutual combination, found on gene *MC*1R explain the variation in PC1. Inset window on the graph is useful to interpret the number of samples for each point. *y* = 0.632 *x* + 6.196; *R*_2_ = 0.11.

The PCA on genetic data provided clear separation between wild‐type MC1R and genotypes with ‘domestic’ signature (Fig. 2). The regression between GI scores and litter size was highly significant and positive (slope = 0.632, *t* = 4.880, *P* <<0.001).

## Discussion

Current understanding of the long and winding process of animal domestication is growing and we are becoming increasingly cognizant of the importance of introgression between free‐living and managed animals in that process. For example, melanic North American wolves represent a product of past hybridization with domestic dogs and they have risen to high frequency in forested habitats without snow, exhibiting a molecular signature of positive selection (Anderson et al. 2009). Moreover, it was recently demonstrated a genetic adaptations to cold climate in pig populations from high‐latitude Chinese regions, that might have been introgressed from an extinct *Sus* species, providing new insights into the role of introgression in adaptation among pigs species.

The European pig was initially domesticated in the Near East and then subsequently introduced into Europe where they encountered free‐living boar adapted to local conditions. Introgression of traits from managed populations into wild ones is generally deleterious, especially for domestic coat colour traits. According to this hypothesis *MC1R* alleles leading to anything other than wild‐type camouflage coat colour are quickly eliminated in the wild (Fajardo et al. 2008).

This disadvantage has, for instance, been recently argued as a support for the claim that Mesolithic hunters‐gatherer populations in Northern Europe possessed domestic pigs acquired from near‐by farming communities (Zeder 2012; Krause‐Kyora et al. 2013; Evin et al. 2014; Rowley‐Conwy and Zeder 2014). Otherwise, our results suggest that the benefits of introgression between managed and free‐living populations are not a one way street: *good* when going from free‐living to managed, but *bad* the other way around. In our case domestic traits, like spotted coat colour, might make animals more visible and thus more susceptible to human and nonhuman predation, but it may confer other traits too (i.e. increased litter sizes) that might compensate, especially in heterozygous females. We argue that gene flow between domestic and wild forms is thus genuinely advantageous to boars' fertility, even if, prediction about the strength of natural selection on domestic phenotypic traits is complex because of epistatic gene effects, and ontogenetic constraints.

In wild boars examined here, mean litter size is higher than expected by the clinal variation in Eurasia (Bywater et al. 2010) and sows bearing with nonsynonymous mutations have statistically larger litter. In this species, the reproductive rate is significantly influenced by food availability (Gethöffer et al. 2007). Sows maximize their reproductive output by changing the share of resources allocated to offspring production when food is plenty (Gamelon et al. 2014). However, body size in considered sows is unlinked to the number of offspring, or to specific ecological factors.

Reproductive activities in general, and large litter in particular, can increase predation risk in all phases of the breeding events. The principal natural predator of the wild boar, the wolf, is experiencing a significant population growth via recolonization from the East in Italy (Lucchini et al. 2002; Marucco and McIntire 2010) and wolves showed a heavier predation impact in warmer, more productive ecosystem of Southern regions of Europe than elsewhere (Melis et al. 2006). In recent decades, both the population size and range extent of wolves have increased in Italy. Wolves are recolonizing their historical range, moving from the Apennines to the Western part of the Italian Alps (Scandura et al. 2001; Fabbri et al. 2007). In Italian Apennine wolf diet is dominated by boar (61.50 ± 3.90, mean ± SE, % of biomass eaten), with high proportion of piglets >77% (Mattioli et al. 1995). These evidences suggest that large litters and fast genetic evolution of MC1R diversity, in our wild boar, cannot simplistically be explained by the *relaxed selection*.

Considering the wild population and its domestic counterpart as demes of the same metapopulation, we deal with an unusual case of interdemic selection (*sensu*Wright), where differential migration of individuals occurs in demes with high fitness. According to this hypothesis, phases I and II of the Wright's interdemic selection are represented by the domestication process in pig and by natural selection in wild boar. Migration and differential interdemic selection between the two forms create new adaptive gene combinations; some of them positively selected and attracted to different fitness peaks, becoming more and more genetically different from each other.

Interdemic selection represents somewhat of a departure from the traditional notions of natural selection. It operates on the genetic variance among demes, rather than that among individuals. The population at large or ‘metapopulation’ consists of an array of demes, each of which may expand, contract, become extinct and contribute to recolonization.

Here, we do not observe a case of spatially spread conventional metapopulation because our demes are represented by ‘domestic’ and ‘wild’ systems that in some cases can be sympatric although genetically distinct.

The interdemic selection as been supported almost entirely by single proponent V.C. Wynne Edwards (Wynne Edwards 1962) and the conditions under which it could operate were thought to be too restrictive for confirm them in natural populations. In effect, during the subsequent period we were aware of only few studies (Mallet and Singer 1987; Smith and Hagen 1996; Avilés 1997; Goodnight and Stevens 1997; Wade and Goodnight 1998).

In our case, both demes have high fitness in their environments. In pigs, parameters for reproduction and production are heritable (Onteru et al. 2012), probably because genes implied in reproduction experienced a differential dispersion (phase III migration in Wright's model). Although there could be a bidirectional gene flow between the two populations, we considered here only the effect of migration towards wild boar.

The increase in genetic diversity of a natural population by an artificially selected mutation may provide a source of variability for adaptation, as the case of black wolf (Anderson et al. 2009).

Our inferences are based on 62 pregnant sows, because obtaining a large data set of this species is somewhat difficult. Nevertheless, our results strongly supported the hypothesis of positive influence of increased fertility from domestic pigs towards wild individuals, even if they can also be understood in terms of individual‐level selection. It would be welcome assembling a larger data set in order to provide more information on this unusual, yet interesting phenomenon.

Our observation has relevance not only for understanding initial domestication, but also for continuing issues of crosses between domestic and free‐living animals and, possibly, for attempts to reintroduce endangered animals subject to captive breeding programs into the wild.

Moreover, we should consider that the wild boar is one of the most widespread and invasive species around the world. Our study in evolutionary application topics about hybrid populations can help to better understand the factors that may determine their invasive potential and to guide future study and control efforts.

Hybridized populations can experience an increase of local adaptation to environmental variation and this is difficult to test in controlled experimental settings on large mammals like wild boar.

Even if more studies on this phenomenon are still required, this research can be useful to policy makers to classify the protection status or to conduct management practices for wild populations of high ecological and economical value.

## Data Archiving Statement

Data available from the Dryad Digital Repository: http://dx.doi.org/10.5061/dryad.c96j0.

## Ethics statement

Authors confirm that the manuscript has not been submitted elsewhere, and that all research meets the ethical guidelines of Italy. Samples were collected during legal hunts in accordance with Italian National laws (*157/92 and 394/91 Laws*) and all experimental protocol were approved by the Ministry of Environment (ISPRA, prot n 24581 20/07/2014).

## Conflict of Interest

The authors declare no conflict of interests.

## Supporting information


**Table S1.** Genetic and morphological characteristic of pregnant wild boar samples collected.
