# Supplementary material for: Unexpected but welcome. Artificially selected traits may increase fitness in wild boar
Source: Evol Appl. 2016 May 17;9(6):769–76. doi: 10.1111/eva.12383 (PMC4908463; doi:10.1111/eva.12383)
Supplement: Supplementary file 1 — Table S1. Genetic and morphological characteristic of pregnant wild boar samples collected. [file EVA-9-769-s001.doc]

**Supplementary Table S1: Genetic and morphological characteristic of pregnant wild boar samples collected.**

| Sample | Wild type | Synonymous substitutions | Non-synonymous substitutions | Number of foetus | Weight (kg) | Type of substitution (O homozygosity; E heterozygosity) | Coat Color | Type | MC1R GeneBank accession number | mtDNA GeneBank accession number |
| --- | --- | --- | --- | --- | --- | --- | --- | --- | --- | --- |
| FM1 | 0 | 0 | 1 | 8 | 52 | E | wild type | 6 | KP984774 | HM51199 |
| FM2 | 0 | 0 | 1 | 10 | 75 | E | wild type | 6 | KP984774 | HM51200 |
| FM3 | 0 | 0 | 1 | 6 | 80 | E | wild type | 6 | KP984774 | HM051193 |
| FM4 | 0 | 0 | 1 | 8 | 79 | E | wild type | 7 | KP984775 | HM051193 |
| FM5 | 0 | 0 | 1 | 10 | 80 | O | dominant black | 8 | KP984776 | HM51199 |
| FM6 | 0 | 0 | 1 | 8 | 80 | O | dominant black | 8 | KP984776 | KF873479 |
| FM7 | 0 | 0 | 1 | 10 | 80 | E | wild type | 9 | KP984777 | HM51200 |
| FM8 | 0 | 0 | 1 | 4 | 47 | E | wild type | 9 | KP984777 | KR865953 |
| FM9 | 0 | 0 | 1 | 8 | 68 | E | wild type | 9 | KP984777 | HM051193 |
| FM10 | 0 | 0 | 1 | 7 | 56 | E | wild type | 9 | KP984777 | HM51194 |
| FM11 | 0 | 0 | 1 | 8 | 53 | E | wild type | 9 | KP984777 | HM051193 |
| FM12 | 0 | 0 | 1 | 10 | 75 | E | wild type | 10 | KP984778 | HM51200 |
| FM13 | 0 | 0 | 1 | 8 | 54 | deletion | wild type | 13 | KP984781 | HM051193 |
| FM14 | 0 | 1 | 0 | 6 | 40 | O | wild type | 1 | KP984769 | HM51200 |
| FM15 | 0 | 1 | 0 | 6 | 42 | O | wild type | 1 | KP984769 | HM051193 |
| FM16 | 0 | 1 | 0 | 6 | 70 | O | wild type | 1 | KP984769 | HM51200 |
| FM17 | 0 | 1 | 0 | 6 | 68 | O | wild type | 1 | KP984769 | HM051193 |
| FM18 | 0 | 1 | 0 | 4 | 57 | E | wild type | 2 | KP984770 | HM051193 |
| FM19 | 0 | 1 | 0 | 6 | 40 | O | wild type | 2 | KR865958 | HM051193 |
| FM20 | 0 | 1 | 1 | 8 | 79 | insertion | wild type | 4 | KP984772 | HM051193 |
| FM21 | 0 | 1 | 1 | 6 | 75 | O, O | wild type | 5 | KP984773 | HM51200 |
| FM22 | 0 | 1 | 1 | 6 | 58 | E, E | wild type | 11 | KP984779 | KF873478 |
| FM23 | 0 | 2 | 0 | 10 | 88 | O, E | black spotted | 3 | KP984771 | HM448432 |
| FM24 | 0 | 2 | 1 | 7 | 55 | E, E, E | wild type | 12 | KP984780 | HM051193 |
| FM25 | 1 | 0 | 0 | 6 | 60 |  | wild type |  | KF780580 | KF873479 |
| FM26 | 1 | 0 | 0 | 5 | 60 |  | wild type |  | KF780580 | KF873479 |
| FM27 | 1 | 0 | 0 | 4 | 55 |  | wild type |  | KF780580 | HM51200 |
| FM28 | 1 | 0 | 0 | 4 | 55 |  | wild type |  | KF780580 | HM51200 |
| FM29 | 1 | 0 | 0 | 5 | 70 |  | wild type |  | KF780580 | HM51200 |
| FM30 | 1 | 0 | 0 | 4 | 55 |  | wild type |  | KF780580 | HM51194 |
| FM31 | 1 | 0 | 0 | 5 | 38 |  | wild type |  | KF780580 | HM051193 |
| FM32 | 1 | 0 | 0 | 5 | 56 |  | wild type |  | KF780580 | HM051193 |
| FM33 | 1 | 0 | 0 | 5 | 43 |  | wild type |  | KF780580 | KR865954 |
| FM34 | 1 | 0 | 0 | 5 | 60 |  | wild type |  | KF780580 | HM51200 |
| FM35 | 1 | 0 | 0 | 5 | 65 |  | wild type |  | KF780580 | HM051193 |
| FM36 | 1 | 0 | 0 | 5 | 50 |  | wild type |  | KF780580 | HM51200 |
| FM37 | 1 | 0 | 0 | 5 | 56 |  | wild type |  | KF780580 | HM51200 |
| FM38 | 1 | 0 | 0 | 4 | 48 |  | wild type |  | KF780580 | HM51200 |
| FM39 | 1 | 0 | 0 | 6 | 55 |  | wild type |  | KF780580 | HM51200 |
| FM40 | 1 | 0 | 0 | 5 | 57 |  | wild type |  | KF780580 | HM51200 |
| FM41 | 1 | 0 | 0 | 5 | 66 |  | wild type |  | KF780580 | KR865955 |
| FM42 | 1 | 0 | 0 | 7 | 65 |  | wild type |  | KF780580 | HM051193 |
| FM43 | 1 | 0 | 0 | 6 | 58 |  | wild type |  | KF780580 | HM051193 |
| FM44 | 1 | 0 | 0 | 7 | 45 |  | wild type |  | KF780580 | HM51199 |
| FM45 | 1 | 0 | 0 | 8 | 60 |  | wild type |  | KF780580 | HM051193 |
| FM46 | 1 | 0 | 0 | 6 | 25 |  | wild type |  | KF780580 | HM051193 |
| FM47 | 1 | 0 | 0 | 4 | 23 |  | wild type |  | KF780580 | KF873475 |
| FM48 | 1 | 0 | 0 | 5 | 54 |  | wild type |  | KF780580 | HM051193 |
| FM49 | 1 | 0 | 0 | 8 | 58 |  | wild type |  | KF780580 | KF873475 |
| FM50 | 1 | 0 | 0 | 4 | 61 |  | wild type |  | KF780580 | KF873475 |
| FM51 | 1 | 0 | 0 | 3 | 52 |  | wild type |  | KF780580 | HM51194 |
| FM52 | 1 | 0 | 0 | 6 | 57 |  | wild type |  | KF780580 | KR865956 |
| FM53 | 1 | 0 | 0 | 6 | 60 |  | wild type |  | KF780580 | KR865956 |
| FM54 | 1 | 0 | 0 | 6 | 73 |  | wild type |  | KF780580 | HM051193 |
| FM55 | 1 | 0 | 0 | 6 | 63 |  | wild type |  | KF780580 | HM51200 |
| FM56 | 1 | 0 | 0 | 6 | 60 |  | wild type |  | KF780580 | HM051193 |
| FM57 | 1 | 0 | 0 | 5 | 60 |  | wild type |  | KF780580 | HM051193 |
| FM58 | 1 | 0 | 0 | 6 | 57 |  | wild type |  | KF780580 | KF873473 |
| FM59 | 1 | 0 | 0 | 6 | 65 |  | wild type |  | KF780580 | KR865957 |
| FM60 | 1 | 0 | 0 | 6 | 55 |  | wild type |  | KF780580 | KF873479 |
| FM61 | 1 | 0 | 0 | 6 | 54 |  | wild type |  | KF780580 | KF873479 |
| FM62 | 1 | 0 | 0 | 6 | 52 |  | wild type |  | KF780580 | KF873479 |
